# Fluid responsiveness in acute circulatory failure

**DOI:** 10.1186/s40560-015-0117-0

**Published:** 2015-11-19

**Authors:** Ahmed Hasanin

**Affiliations:** Department of Anesthesia and Critical Care Medicine, Cairo University, Giza, Egypt

**Keywords:** Fluid responsiveness, Acute circulatory failure, Heart-lung interaction, Fluid challenge

## Abstract

Although fluid resuscitation of patients having acute circulatory failure is essential, avoiding unnecessary administration of fluids in these patients is also important. Fluid responsiveness (FR) is defined as the ability of the left ventricle to increase its stroke volume (SV) in response to fluid administration. The objective of this review is to provide the recent advances in the detection of FR and simplify the physiological basis, advantages, disadvantages, and cut-off values for each method. This review also highlights the present gaps in literature and provides future thoughts in the field of FR.

Static methods are generally not recommended for the assessment of FR. Dynamic methods for the assessment of FR depend on heart-lung interactions. Pulse pressure variation (PPV) and stroke volume variation (SVV) are the most famous dynamic measures. Less-invasive dynamic parameters include plethysmographic-derived parameters, variation in blood flow in large arteries, and variation in the diameters of central veins. Dynamic methods for the assessment of FR have many limitations; the most important limitation is spontaneous breathing activity.

Fluid challenge techniques were able to overcome most of the limitations of the dynamic methods. Passive leg raising is the most popular fluid challenge method. More simple techniques have been recently introduced such as the mini-fluid challenge and 10-s fluid challenge. The main limitation of fluid challenge techniques is the need to trace the effect of the fluid challenges on SV (or any of its derivatives) using a real-time monitor. More research is needed in the field of FR taking into consideration not only the accuracy of the method but also the ease of implementation, the applicability on a wider range of patients, the time needed to apply each method, and the feasibility of its application by acute care physicians with moderate and low experience.

## Introduction

Fluid resuscitation is the cornerstone of managing patients having acute circulatory failure. Although restoring the volume status of a shocked patient is substantial, growing evidence indicates that unnecessary administration of fluids has a deleterious outcome [1].

Fluid responsiveness (FR) is defined as the ability of the left ventricle to increase its stroke volume (SV) in response to fluid administration [2]. Assessment of patient response to volume expansion presents a daily challenge for acute care physicians. FR has been extensively evaluated in various situations with acute circulatory failure such as septic shock, cardiac surgery, and other surgical procedures.

The objective of this review is to provide the recent advances in the detection of FR and simplify the physiological basis, advantages, disadvantages, and cut-off values for each method. The review also highlights the present gaps in literature and provides future thoughts in the field of FR.

The review is organized into the following sections:

1. Static parameters and its disadvantages
2. Dynamic parameters: physiological basis, different varieties, and limitations
3. Fluid challenge methods: physiological basis, different varieties, and limitations
4. Present gaps in the literature, conclusions, and future thoughts

## Review

### Static measures to assess FR

#### Measures

##### *Pressures*

Both central venous pressure (CVP) and pulmonary artery occlusion pressure (PAOP) were reported to be of a poor value in prediction of FR in both spontaneously breathing [3] and mechanically ventilated patients [4–6].

##### *Areas and volumes*

Although they were considered to be good indicators of preload [7], cardiac dimensions (left ventricular end diastolic area (LVEDA) and left ventricular end diastolic volumes (LVEDV)) were reported as poor predictors of FR [3, 6, 8].

##### *Diameters*

Inferior vena cava (IVC) maximum diameter (measured by ultrasound at the subcostal area) [9, 10] and vascular pedicle width (measured from chest X-ray) [11, 12] have gained popularity in the assessment of volume status. Moderate evidence is available suggesting that IVC diameter is lower in hypovolemic patients [9, 10]. The role of vascular pedicle width is more evident in patients with volume overload [12, 13]. There is no evidence suggesting the role of absolute IVC diameter or vascular pedicle width in the prediction of FR in shocked patients.

#### Limitations of static methods

Many reasons described why static parameters are of poor value in the prediction of FR. First, cardiac filling pressures represent intramural pressure; however, preload is determined by transmural pressure, which is affected by both intramural and extramural pressures [14]. Second, preload alone cannot predict FR because the response of a patient to fluids depends on both preload and cardiac contractility that varies between patients. Preload will predict FR only in cases with normal ventricular contractility [15].

### Dynamic measures to assess FR

#### Physiological basis

FR is detected by inducing a change in the preload and subsequently monitoring the corresponding change in SV or one of its derivatives. This change in preload is achieved in dynamic measures by positive pressure ventilation. Positive pressure ventilation provokes a cyclic decrease in the right ventricular (RV) SV via two mechanisms:

1. Decreased preload (decreased venous return)
2. Increased afterload (increased transpulmonary pressure) [16].

RV stroke volume reaches its minimum value by the end of inspiration resulting in a consequent decrease in LV filling and thus LV stroke volume after a lag period of 2–3 heartbeats [17, 18].

Two other mechanisms were mentioned to antagonize the negative effect of mechanical ventilation on SV:

1. Squeezing of blood out of alveolar vessels and thus transiently increasing LV preload [19]
2. Inspiratory increase in pleural pressure that decreases LV afterload, enhancing LV ejection

However, experimental data suggest that the latter two mechanisms have a minor effect on LV stroke volume. Consequently, the net effect of positive pressure ventilation is decreasing RV stroke volume, LV filling, and LV stroke volume [16]. This decrease in SV is more prominent when the patient is on the steep part of cardiac contractility “Frank-Starling” curve (fluid responder).

#### Measures

##### *Systolic pressure variation (SPV)*

SPV is calculated by measuring the difference between both the maximal and the minimal values of systolic blood pressure (SBP) during a single respiratory cycle and a reference value (reference value is SBP measured at end expiratory pause) [16]. SPV was reported to increase with induction of hypovolemia in mechanically ventilated dogs [20], mechanically ventilated patients after aortic surgery [21], and patients with septic shock [22]. Denault et al. [23] reported SPV to be affected by airway and pleural pressures rather than volume status; however, a meta-analysis conducted by Marik et al. showed area under receiver operating characteristics (AUROC) = 0.86 for SPV for the detection of FR [6].

##### *Pulse pressure variation (PPV)*

PPV is calculated by dividing the largest PP (PP_max_ − PP_min_) by the average PP (PP_max_ + PP_min_/2). PPV above 13 % [24] is a good predictor of FR [25] [6]. AUROC was reported as 0.98 in a prospective cohort study [26] and 0.94 in a meta-analysis [6]. PPV has an advantage over SPV of not being affected by airway and pleural pressure because these pressures affect both SBP and diastolic blood pressure (DBP), whereby the PP (difference between SBP and DBP) remains unaffected [27]. In the intraoperative situation, PPV showed low accuracy with patients undergoing gastrointestinal (GIT) surgery [28]. PPV was accurate after cardiac surgery only when patients with low perfusion or right ventricular dysfunction were excluded [29].

##### *Stroke volume variation (SVV)*

SVV is commonly measured by PiCCO continuous cardiac output monitoring [30] or esophageal Doppler [31, 32]. It is calculated by dividing the difference between maximum SV and minimum SV (SV_max_ − SV_min_) by their average (SV_max_ + SV_min_/2) in a time window of 30 s. SVV was reported as a good predictor of FR in brain surgery patients [30], general surgical patients [32], septic shock patients [33], and after cardiac surgery [34]. SVV was not accurate with patients undergoing GIT surgery [28]. A recent meta-analysis reported SVV as a reliable predictor for FR in mechanically ventilated patients on tidal volume above 8 ml/kg with AUROC 0.84 [6]. The proper cut-off value was 14 % for SVV [32, 35] and 11 % for stroke output index [31].

##### *Plethysmographic dynamic indices*

Pulse oximetry plethysmographic waveform amplitude (POP) is measured using a special pulse oximetry sensor; plethysmographic variability index (PVI) is more easily measured using Masimo device. Both POP and PVI were reported in a recent meta-analysis as good indicators for FR in mechanically ventilated patients without cardiac arrhythmias, heart failure, or spontaneous activity with cut-off value 9.1–15 % [36]. PVI measured before induction of anesthesia predicted propofol-induced hypotension during induction of anesthesia [37]. PVI showed low accuracy in the detection of FR after cardiac surgery [29].

##### *IVC respiratory variation*

IVC variation is best assessed using ultrasound in the long-axis (sagittal) view. IVC diameter is measured 1 cm distal to its junction with hepatic vein either by 2-D or M modes [38]. Although data about IVC variation in FR are heterogeneous with regard to the type of patients, type of fluid, and definition of collapsibility index, there is a consensus that a cut-off value of 12–21 % for IVC variation is useful in the detection of FR in mechanically ventilated patients [39, 40]. In spontaneously breathing patients, a more cautious use was suggested by Muller and co-workers who reported IVC variability >40 % to predict FR. They also suggested that low values (<40 %) do not exclude FR [41].

##### *SVC respiratory variation*

SVC variation (measured using TEE) predicts FR with a cut-off value that ranged between 29 [40] and 36 % [42]. Variation in the peak flow velocity in SVC predicts FR at a cut-off value of 12.7 % [43].

##### *Internal jugular vein (IJV) and subclavian vein respiratory variation*

IJV distensibility measured using ultrasound was recently reported as a predictor of FR in mechanically ventilated patients with cut-off value 18 %. The combination of IJV distensibility of 9.7 % and PPV more than 12 % reaches a sensitivity of 100 % and specificity of 95 % [44]. A recent study reported IJV collapsibility index to overestimate the collapsibility when compared with IVC; they also reported a weak correlation between IJV and IVC collapsibility, raising a controversy about IJV use in the prediction of FR [45]. Subclavian vein collapsibility was also reported as an alternative method to detect FR with the advantage of having easy and fast access than IVC [46].

##### *Subaortic velocity time integral (VTI) variation*

Subaortic VTI variation was reported by Slama et al. as a predictor for FR in experimental animals [47]. However, it has not been tried in humans yet.

##### *Aortic velocity variation*

Respiratory variation of peak aortic velocity was reported as a good indicator for FR in critically ill patients under mechanical ventilation: the best cut-off value was 18 % when measured by esophageal Doppler [48] and 12 % when measured by trans-esophageal echocardiography [49]. In a study conducted by Guinot et al., peak aortic velocity measured by esophageal Doppler did not predict FR during surgery [32].

##### *Carotid artery peak velocity variation*

Carotid artery peak velocity variation measured using ultrasound was recently reported in two observational studies to predict FR in patients undergoing coronary artery bypass grafting (CABG) surgery [50] as well as patients with septic shock [51] with cut-off values 11 and 14 %, respectively.

A comparison between different dynamic methods is presented in Table 1.

**Table 1:** Dynamic methods for the detection of FR

Parameter	Cut-off value (%)	Evidence	Limitations
SPV [6, 16, 20–23]	NA	Cohort	Affected by airway and pleural pressure [17]
PPV [6, 24–29]	13	Meta-analysis	Needs special monitor; needs arterial line
SVV [6, 30, 32–35]	14	Cohort	Needs either arterial line (plus special monitor) or esophageal Doppler
SOI [31]	11	Cohort	Needs esophageal Doppler
POP [36]	9.5–15	Meta-analysis	Needs special pulse oximeter
PVI [36, 37]	9.5–15	Meta-analysis	Needs Masimo device
IVC variation [38–41]	12–21	Meta-analysis	Needs ultrasound; difficult in abdominal surgical cases; cannot be used intraoperatively
SVC variation [40–42]	29–36	Cohort	Needs trans-esophageal echocardiography
IJV variation [44, 45]	18	Cohort	Needs ultrasound
Subaortic VTI [47]	NA	Animal study	Needs echocardiography; needs good operator experience; not tried in humans yet
Aortic velocity variation [48, 49]	12–18	Cohort	Needs echocardiography or esophageal Doppler
Carotid velocity variation [50, 51]	11–14	Cohort	Needs ultrasound

*SPV* systolic pressure variation, *PPV* pulse pressure variation, *SVV* stroke volume variation, *SOI* stroke output index, *POP* pulse oximetry plethysmographic waveform amplitude, *PVI* Plethysmographic variability index, *IVC* inferior vena cava, *SVC* superior vena cava, *IJV* internal jugular vein, *VTI* velocity time integral, *NA* not available

#### Limitation of dynamic measures

##### *Spontaneous breathing*

Spontaneous breathing is the most important limitation of dynamic methods. Failure of dynamic methods in detecting FR was reported in patients with spontaneous breathing [52, 53] as well as patients on pressure support ventilation [54]. This was explained by the dependence of dynamic parameters on regular variations in intrathoracic pressure, tidal volume, and rate; all these components are highly variable in spontaneous breathing patients in addition to the effect of abdominal muscle contractions (which is common with spontaneous breathing efforts) on the preload response [55].

##### *Ventilator-related causes*

Dynamic parameters poorly detect FR with tidal volumes below 8 ml/kg [56, 57] and patients with low airway driving pressure below 20 cm/H_2_O [58]. This was explained by the fact that low tidal volume induces small variation in thoracic pressure and consequently small changes in preload [59]. With tidal volumes below 8 ml/kg, FR is present even with lower PPV. The cut-off value for prediction of FR with low tidal volumes should be 8 % and not 12 % as in higher tidal volumes [56]. Failure of dynamic methods was also reported in cases with high-frequency ventilation; this is because the low number of cardiac cycles per respiratory cycle will not allow respiratory variation in SV to occur [60].

##### *Low lung compliance*

Dynamic methods for the detection of FR were reported to be of low value in cases of reduced lung compliance, especially in cases of low PPV [26, 59, 61]; however, the presence of high PPV in patients of acute respiratory distress syndrome (ARDS) is an indicator of FR when patient tidal volume is above 8 ml/kg [59].

##### *Elevated pulmonary artery pressure*

PPV poorly predicted FR in patients with elevated pulmonary artery pressure. These patients usually do not respond to fluid resuscitation [62].

##### *Cardiac causes*

Dynamic measures are not valid in cases having cardiac arrhythmias [63], and variation in SV in these cases is influenced by irregular rhythm rather than heart-lung interactions. PPV was not able to predict FR in patients with elevated LV pressure (defined as E/E’ > 15) [64]. Both PVV and SVV were altered by propranolol-induced acute ventricular failure in mongrel dogs [65].

##### *Open-chest conditions*

Neither PPV nor SVV was able to predict FR under open-chest conditions; this was reported in patients undergoing CABG surgery during thoracotomy. Dynamic methods showed good performance in the same patient group after chest closure [66].

##### *Intra-abdominal hypertension*

Increased intra-abdominal pressure above 10.5 mmHg impairs the accuracy of PPV [67].

A 1-day prospective multicenter study reported that only 2 % of patients with acute circulatory failure in the ICU have fulfilled the validity criteria for valid application of PPV. This finding increases the interest in fluid challenge methods to overcome the known limitations for dynamic methods especially outside the operating room [68].

### Preload and fluid challenge

#### Physiological basis

Fluid challenge methods are done by administration of a fluid bolus (either extrinsically or intrinsically) and monitoring the resulting effect on SV or cardiac output (CO). Fluid responders are patients with 15 % increase in SV or CO after fluid challenge [25]. SV and CO can be measured using transpulmonary thermodilution, pulse contour analysis [25], bioreactance [69], and echocardiography (through measuring subaortic VTI) [25]. Alternative parameters to detect patient response to passive leg raising (PLR) include the following: decreased PPV and SVV [70], increased radial pulse pressure (by 10 %), femoral artery peak velocity [71], carotid artery diameter, and peak velocity [69].

Recently, indirect methods of measurement of the CO response to PLR were introduced. Those include end-tidal CO_2_ (5 % increase) [72], mixed venous oxygen saturation (2 % increase) [73], sublingual microcirculatory perfusion, and skin perfusion [74].

#### Measures

##### *Passive leg raising*

PLR creates a transient increase in the preload via translocation of venous blood from the lower limbs to the thorax. It has been described as an attractive method of fluid challenge having the advantage of being a “self-volume challenge” and a “reversible fluid challenge” [53]. PLR-induced increase in SV (or its derivatives) can determine FR in most situations that dynamic methods fail to deal with such as spontaneous breathing, decreased respiratory compliance, and cardiac arrhythmias [26, 75]. The best cut-off value for detecting FR is increased in aortic blood flow by 10 % (using esophageal Doppler) [53] or increased cardiac index by 10 % (using thermodilution) [26] after PLR.

##### *End expiratory occlusion*

EEO is done by interrupting mechanical ventilation at the end expiratory pause for 15 s with monitoring SV or its derivatives [76]. EEO is considered another method for “self-fluid challenge” as it attenuates the inspiratory increase in thoracic pressure resulting in increasing the venous return [76]. EEO-induced increase in CO by 5 % was reported to predict FR in most patients in whom dynamic measures fail such as patients with arrhythmias [76], decreased respiratory compliance [26], and ARDS patients [77]. EEO can be used in patients with spontaneous breathing activity unless excessive triggering results into test interruption [78].

EEO showed the same accuracy as PLR when the response to both of them was assessed using cardiac index. EEO was superior to PLR if the response was assessed by changes in arterial pulse pressure; this was explained by PLR-induced change in arterial compliance, which impairs the ability of arterial pulse pressure to reflect the changes in SV with PLR [76]. However, PLR has the advantage over EEO in being applicable in non-intubated patients [76]. Although Guinot et al. reported a poor value for EEO in the prediction of FR in the operating theater, this finding was reported in operated patients with no signs of shock [79].

##### *PEEP-induced hemodynamic changes*

Increasing PEEP by 10 cm H_2_O produces a hemodynamic effect that is near to EEO. Geerts et al. reported that FR can be predicted in cardiac surgery patients if increasing PEEP by 10 cm H_2_O for 5 min produced an increase in CVP with AUROC = 0.99; however, no data is available about the cut-off value of the change in CVP that would predict FR [80]. This method has an advantage over EEO of not being affected by ventilator conditions, easily done in ICU patients with no need for any advanced monitors.

Another method for the detection of FR was reported by Wilkman et al. in patients with septic shock by increasing PEEP from 10 to 20 cm H_2_O and following their MAP. A negative predictive value was 100 %, so the absence of decrease of MAP during PEEP elevation can identify non-responders [81].

##### *Arm occlusion pressure*

Arm occlusion pressure is defined as “the radial artery pressure after 35 s occlusion by a blood pressure cuff”; it is measured using an arterial catheter. Arm occlusion pressure is considered as an indicator of mean filling pressure and volume status of the upper limb. Arm occlusion pressure less than 21.9 mmHg detected FR in cardiac surgery patients [82].

##### *Mini-fluid challenge*

Mini-fluid challenge is performed by infusing 100 ml colloids over 1 min with concomitant monitoring of aortic VTI. An increase in VTI by 10 % after mini-fluid challenge predicts FR [83].

##### *Ten-second fluid challenge*

Infusion of 50 ml crystalloids with monitoring of CO or SV was recently reported as a method for the detection of FR. The best cut-off value was 9 % increase in CO or SV after fluid administration [84].

A comparison between different methods for fluid challenge is described in Table 2.

**Table 2:** Fluid challenge methods for the detection of FR

Parameter	Cut-off value	Evidence	Limitations
PLR [26, 53, 75]	10 % increase in aortic flow or CI	Meta-analysis	Not feasible in intraoperative situations and some surgical patients; needs CO monitoring
EEO [26, 76–79]	5 % increase in CO	Cohort	Needs MV; needs CO monitoring
PEEP-induced increase in CVP [80]	1.5 mmHg	Cohort	Tried only in cardiac surgery patients
PEEP-induced decrease in MAP [81]	NA	Cohort	Useful only in identifying non-responders
Arm occlusion pressure [82]	21.9 mmHg	Cohort	Tried only in cardiac surgery patients
Mini-fluid challenge [83]	10 % increase in subaortic VTI	Cohort	Needs echocardiography with experienced operator
10-s fluid challenge [84]	9 % increase in CO or SV	Cohort	Needs CO or SV monitoring

*PLR* passive leg raising, *EEO* end expiratory occlusion, *PEEP* positive end expiratory pressure, *CVP* central venous pressure, *MAP* mean arterial pressure, *CO* cardiac output, *CI* cardiac index, *SV* stroke volume, *BP* blood pressure, *VTI* velocity time integral, *NA* not available

#### Limitations of fluid challenge techniques

The main limitation in using fluid challenge methods for the detection of FR is the need for real-time monitoring of SV or CO to differentiate fluid responders. Alternative less-invasive parameters are being introduced to trace patient response to PLR such as end-tidal CO_2_ [72] and peak velocity in different arteries measured by ultrasound [69, 71].

### Effect of vasopressors on different methods for the detection of FR

Norepinephrine infusion decreases PPV and SVV, masking their ability for the detection of FR. This finding was addressed in two experimental animal studies on dogs [85] and pigs [86]. Moreover, measurement of SV using FloTrac/Vigileo System is not accurate under conditions with increased vasomotor tone due to phenylephrine infusion [87]. On the contrary, the PLR test kept its value as a predictor of FR in patients with septic shock even after increasing norepinephrine dose [88].

### Present gaps in the literature

Four problems with FR still need to be solved:

1. Most of the dynamic methods for the detection of FR need invasive monitoring by an arterial line (except plethysmographic indices and ultrasound indices); less-invasive methods are needed.
2. Most of the fluid challenge techniques need real-time SV monitoring (or VTI monitoring using echocardiography); more simple monitors are needed.
3. Methods suitable for intraoperative use are still limited.
4. The ease of application for any method by a non-expert physician should be considered.

## Conclusions

Static methods are generally not recommended for the detection of FR. Dynamic methods rely on heart-lung interactions. Dynamic methods are accurate and reliable; however, these measures are useful in few selected types of patients. PPV and SVV are the most popular dynamic methods. Further research is needed for introducing less-invasive dynamic methods with fewer limitations.

Fluid challenge methods were able to overcome most of the limitations of dynamic methods; however, these methods need a real-time monitoring for SV or one of its derivatives. PLR is considered to be the standard method for fluid challenge. Recent publications described more simple methods (e.g., 10-s fluid challenge, carotid artery peak velocity variation). These new methods still need more validation in different types of patients. Validation is also needed for more simple methods for tracing SV response to fluids.

Comparing various methods for the detection of FR should take into consideration not only the accuracy of the method but also the ease of implementation, the applicability on a wider range of patients, the time needed to apply each method, and the feasibility of its application by acute care physicians with moderate and low experience.

## Abbreviations

ABP: arterial blood pressure
ARDS: acute respiratory distress syndrome
AUROC: area under receiver operating characteristics
CABG: coronary artery bypass grafting
CO: cardiac output
Co_2_: carbon dioxide
CVP: central venous pressure
DBP: diastolic blood pressure
EEO: end expiratory occlusion
FR: fluid responsiveness
ICU: intensive care unit
IVC: inferior vena cava
LV: left ventricular
LVEDA: left ventricular end diastolic area
LVEDV: left ventricular end diastolic volumes
MAP: mean arterial pressure
PAOP: pulmonary artery occlusion pressure
PEEP: positive end expiratory pressure
PLR: passive leg raising
POP: pulse oximetry plethysmographic waveform amplitude
PPV: pulse pressure variation
PVI: plethysmographic variability index
RV: right ventricular
SBP: systolic blood pressure
SPV: systolic pressure variation
SV: stroke volume
SVC: superior vena cava
Svo_2_: ventral venous oxygen saturation
SVV: stroke volume variation
TEE: trans-esophageal echocardiography
TTE: trans-thoracic echocardiography
VTI: velocity time integral
